# Technological-Based Interventions in Cancer and Factors Associated With the Use of Mobile Digital Wellness and Health Apps Among Cancer Information Seekers: Cross-Sectional Study

**DOI:** 10.2196/63403

**Published:** 2025-02-05

**Authors:** Ogochukwu Juliet Ezeigwe, Kenechukwu Obumneme Samuel Nwosu, Oladipo Kunle Afolayan, Akpevwe Amanda Ojaruega, Jovita Echere, Manali Desai, Modupe Olajumoke Onigbogi, Olajumoke Ope Oladoyin, Nnenna Chioma Okoye, Pierre Fwelo

**Affiliations:** 1 Department of Epidemiology The University of Texas Health Science Center Houston, TX United States; 2 Department of Biostatistics The University of Texas Health Science Center Houston, TX United States; 3 Department of Health Promotion and Behavioral Sciences The University of Texas Health Science Center Houston, TX United States; 4 Department of Pharmaceuticals Gwarinpa General Hospital Abuja Nigeria

**Keywords:** cancer intervention, digital health and wellness apps, cancer management, telehealth, mobile health, mhealth, decision-making, United States, cross-sectional study, adult, logistic regression, regression analysis, digital health, young adult, cancer screening, knowledge seeking, barrier, utilization, engagement, digital health engagement, diversity, cancer information, health seeking behavior, mobile phone

## Abstract

**Background:**

Mobile digital wellness and health apps play a significant role in optimizing health and aiding in cancer management and decision-making.

**Objective:**

This study aims to identify the factors influencing the use of mobile health and wellness apps among cancer information seekers in the United States.

**Methods:**

We conducted a cross-sectional study using data from the Health Information National Trends Survey. Our analysis focused on 4770 participants who sought cancer information. We performed weighted univariate and multivariable logistic regression to determine the association between the use of health and wellness apps and socioeconomic factors, medical history and conditions, and lifestyle and behavioral factors.

**Results:**

A total of 4770 participants who sought cancer information were included in the final analysis. Of these, 80.9% (n=2705) were health and wellness app users, while 19.1% (n=793) were nonusers. In the final adjusted model, participants with household incomes ≥US $50,000 had 49% higher adjusted odds of using these apps than those with incomes <US $50,000 (adjusted odds ratio [aOR]=1.49, 95% CI 1.02-2.14). College graduates and those with higher educational levels were avid users compared to those with a high school diploma or less (aOR=1.87, 95% CI 1.30-2.67). Internet users had over 3 times the odds of using these apps compared to nonusers (aOR=3.28, 95% CI 1.70-6.33). Participants within the age group 18-34 years were 3.70 times more likely (aOR=3.70, 95% CI 1.90-7.23) to use a health and wellness app compared to participants within the age group of 75 years and older.

**Conclusions:**

Age, education, household income, and use of the internet are the major determinants of the adoption of digital health and wellness apps among seekers of cancer information. Hence, public health programs could be directed toward addressing these factors to improve cancer diagnosis, treatment, and management using these apps.

## Introduction

Cancer is a major health problem worldwide and the second leading cause of death in the United States [1]. According to the World Health Organization (WHO), there were about 10 million deaths from cancer in 2020 [2]. According to the National Cancer Institute, in January 2022, about 18.1 million survivors of cancer were estimated in the United States, and by 2032, the number of survivors of cancer would increase to 22.5 million [3]. In the United States, the increasing cancer survival rates can be largely attributed to advances in screening, early diagnoses, and treatment of cancers, as well as a growing and aging US population [4]. Many survivors of cancer tend to seek health information in addition to the information provided by their physicians [5]. A study reported that survivors of cancer seek more information about good diet, exercise, and weight management while undergoing treatment [6]. Nearly 50% of Americans and over 60% of survivors of cancer seek cancer-related information from at least 1 source, including mobile wellness and health apps [5,7-9].

According to WHO, mobile health (mHealth) includes using smartphones, sensors, PDAs, wireless monitoring devices, or other wireless devices for public health and medical practices [10]. The Center for Democracy and Technology categorizes health apps into 4 types: health reference, fitness tracker, diagnostic, and disease management [11]. Mobile health apps are software programs running on smartphones and tablets to promote health and primary disease prevention [12,13]. They are used to oversee, improve, and maintain the health of their users at individual and community levels [14]. Furthermore, these health apps are very useful in facilitating medication adherence, monitoring symptoms, clinical decision-making, and behavioral changes [15-17]. Among patients with cancer, mobile digital health is an important consideration when seeking ways to optimize their mental health [18].

A study has shown that mHealth apps can help in primary prevention, such as screening, as well as early diagnosis, management, survivorship, and end-of-life care among patients with cancer [19]. Another study reported promising use of digital health solutions for promoting and managing the cancer care continuum within a patient-centeredness framework [20]. Furthermore, research has shown that mHealth apps can aid health care providers and patients in cancer diagnosis, managing psychological distress, facilitating follow-up care, devising treatment plans, delivering cancer-related information, promoting drug adherence, and addressing side effects [21].

Socioeconomic variables such as education level and income, marital status, gender, and age, are important predictors of choice of health information source among survivors of cancer in the United States [9]. A study that assessed the disparities in access to mobile health devices and eHealth literacy among survivors of breast cancer found that older age, lack of access to mobile devices, and a lower education level had a lesser association with eHealth literacy [22]. This study also showed that younger women with higher education levels and from less deprived areas were more likely to access smartphones and tablets [22]. Another study also found that mHealth users were more likely to be younger, have higher education, reported excellent health, higher income, and intention to change diet and physical activity [23]. A recent review suggested the positive effect of mHealth apps on health outcomes among those enduring chronic diseases [24]. Furthermore, research revealed that older adults exhibited diminished self-efficacy when using mHealth apps often stemming from a deficiency in technical skills and resulting in a decreased inclination to engage with the technology [25]. An analysis of the moderating effect of different age groups suggests that the perceived ease of use and vulnerability were associated with the use of mHealth apps among middle-aged and elderly people [26].

Despite the growth and promise of using mobile apps to deliver information and interventions to patients with cancer and those with other chronic diseases, the factors influencing the use of mHealth apps have been well-studied primarily among survivors of cancer. However, there is limited research on these factors among the broader US population seeking cancer information. Therefore, the purpose of this study is to identify the factors that impact the usage of health and wellness apps among those seeking cancer information here in the United States. By using a sequential modeling approach, this study aims to provide a deeper understanding of the factors associated with mobile app usage in this specific population, ultimately informing targeted interventions and strategies to effectively support cancer information seekers, both survivors and nonsurvivors.

## Methods

### Data Source, Study Design, and Setting

This cross-sectional study analyzed data from the Health Information National Trends Survey (HINTS), a nationwide representative survey of US adults aged 18 years and older in 2020 and 2022. HINTS has been conducted periodically since 2003. HINTS collects information on access to and usage of health-related information, health-related behaviors such as perceptions, knowledge of disease and cancer screening as well as telehealth among US adults. The HINTS uses a complex sampling design to ensure the representativeness of the adult population in the United States. The survey uses a sampling frame provided by Marketing Systems Group of addresses in the United States. To enhance the response rate and ensure the sample’s representativeness, HINTS conducts several follow-up mailings for nonrespondents. Detailed information about the methodology, sampling, and weighting is available on the web [27]. This study is reported following the STROBE (Strengthening the Reporting of Observational Studies in Epidemiology) guidelines [28].

### Study Population

This study included adults aged 18 and older in the United States. Using consistent weighting algorithms, we merged data from HINTS 5 (Cycle 4, 2020) and HINTS 6 (2022) to increase the precision of estimates and power of this study. This resulted in a total population of 10,117 respondents from the 2 surveys. Individuals’ cancer-seeking behavior was assessed through a single question: Have you ever looked for information about cancer from any source? The response options were “yes” or “no.” Those who answered “yes” were classified as having sought cancer-related information. Hence, this study included 4770 participants who actively sought cancer-related information in the analysis.

### Study Variables

#### Outcome

The outcome variable was using health and wellness apps categorized as “yes” or “no.” Two sequential survey questions were used depending on the outcome. Participants were asked if they owned tablets, computers, or smartphones. Those who owned any were further asked if they had health and wellness apps on these devices, with response options of “yes,” “no,” or “do not know.” If they answered “yes,” they were then asked if they had used these apps in the past 12 months.

#### Independent Variables

Based on previous literature, the sociodemographic characteristics: age (18-34, 35-49, 50-64, 65-74, or 75 years and older), gender at birth (male or female), insurance (yes or no), educational level (high school or less, some college, or college graduates and more), household income (<US $50,000 or ≥US $50,000), use of the internet (yes or no) and race or ethnicity (Hispanic, non-Hispanic Asian or non-Hispanic other, non-Hispanic Black, or non-Hispanic White); medical history and disease conditions: presence or absence of cancer (yes or no), general health status (fair or poor, good, or excellent and very good), family history of cancer (yes or no), depression (yes or no), diabetes (yes or no), hypertension (yes or no), heart condition (yes or no), chronic lung disease (yes or no), and number of disease condition (none, one, or two or more), and lifestyle or behavioral characteristics; BMI (underweight, normal weight, overweight, or obese) and physical activity were included in this study. Physical activity was assessed by the number of days per week and duration of moderate-intensity exercise, classified based on the WHO’s recommendation of 150 minutes/week [9,22-27].

#### Statistical Analysis

We used weighted frequencies and percentages to present participants’ sociodemographic characteristics, medical history, disease conditions, lifestyle, and behavioral traits by mobile health app usage status. We used the Pearson chi-square test to assess the statistical significance (*P*<.05) of the relationship between mobile health and wellness app usage and independent variables. We conducted bivariate and multivariable logistic regression analyses to evaluate the association between the outcome and covariates.

We fitted 4 sequential modeling approaches in the multivariable analysis. Model 0 included the crude effects of each covariate on health and wellness app usage. Model 1 incorporated the effects of sociodemographic characteristics on health and wellness app usage. Model 2 adjusted for all covariates from model 1 and medical history and disease conditions, such as cancer, general health status, family history of cancer, depression, diabetes, hypertension, heart conditions, chronic lung disease, and the number of chronic diseases. The final (model 3) included model 2 and lifestyle and behavioral characteristics, such as BMI and physical activities.

This sequential modeling strategy aimed at determining the degree to which mHealth app use was explained by each group of variables among seekers of cancer information. We further explored the relative importance of medical history and disease conditions on sociodemographic factors and how medical disease conditions are related to the use of mobile apps (model 2). We also looked at the specific importance behavior and lifestyle have in the final model (model 3). For this study, we chose to adjust for physical activity and BMI because these variables are potential factors for the usage of wearable devices [29,30].

All statistical analyses were performed using SAS software (version 9.4; SAS Institute), with significance set at a *P* value <.05 and a 95% CI.

### Ethical Considerations

The administration of HINTS received approval from the institutional review board at Westat Inc and was designated as exempt by the National Institutes of Health Office of Human Subjects Research (45 CFR 46.104 and Project # 6632.03.51) [27]. This exemption applies to this study. HINTS data are accessible to the public, and additional information regarding the survey methodology is available on the HINTS website. This study’s participants were deidentified and there was no patient contact.

## Results

A total of 4770 participants were seeking cancer information. Only 3498 participants had complete information on the use of mobile apps. Of these, 80.9% (n=2705) were health and wellness app users, while 19.1% (n=793) were nonusers. Overall, more than half (n=2894, 58.4%) of the participants were female, and 78% were between the age group 18-64 years. A larger percentage of participants were college graduates (n=2606, 41.5%), identified as non-Hispanic White (n=2936, 69.3%), had a household income exceeding US $50,000 (n=2854, 69.9%), and rated their general health as excellent or good (n=2256, 49.1%). Most participants had no history of cancer (n=3538, 84.7%), had insurance (n=4453, 92.1%), were internet users (n=4376, 93.3%), had heart conditions (n=460, 92.4%), and lung diseases (n=3961, 86.7%; Table 1).

**Table 1:** Distribution of participants characteristics by the use of wellness and health apps (N=4770).

Characteristic					Seekers of cancer information (N=4770), n^a^ (Wt%)^b^			Used health and wellness app (n=3498)						
								Yes, n^a^ (Wt%)^b^			No, n^a^ (Wt%)^b^			*P* value
**Used health and wellness apps**														
	Yes			2705 (80.9)			—^c^			—			—	
	No			793 (19.1)			—			—			—	
**Sociodemographic and economic characteristics**														
	**Age (years)**												<.001	
		18-34	643 (21.8)			491 (27)			73 (13.5)					
		35-49	931 (27.1)			658 (28.7)			111 (22.9)					
		50-64	1429 (29.1)			810 (29.4)			251 (34.9)					
		65-75	1110 (13.9)			512 (10.6)			237 (19.4)					
		75+	575 (7.2)			207 (4.3)			107 (9.4)					
	**Gender at birth**												.18	
		Female	2894 (58.4)			1693 (59.9)			462 (55.8)					
		Male	1683 (41.6)			893 (40.1)			299 (44.2)					
	**Insurance**												.82	
		Yes	4453 (92.1)			2548 (92.9)			724 (92.6)					
		No	239 (7.9)			118 (7.04)			54 (7.4)					
	**Education**												<.001	
		High school or less	1015 (27.3)			410 (21.8)			219 (33.3)					
		Some college	950 (31.2)			510 (30)			178 (34.2)					
		College graduates and more	2606 (41.5)			1666 (48.2)			367 (32.6)					
	**Race or ethnicity**												.43	
		Hispanic	597 (12.9)			324 (12.6)			110 (11.4)					
		Non-Hispanic Asian or non-Hispanic other	332 (8.9)			201 (9.6)			59 (7.1)					
		Non-Hispanic Black	517 (8.9)			296 (8.3)			86 (8.3)					
		Non-Hispanic White	2936 (69.3)			1700 (69.6)			467 (73.3)					
	**Household income (US $)**												.001	
		<50,000	1456 (30.1)			628 (22.8)			273 (33.6)					
		≥50,000	2854 (69.9)			1853 (77.2)			437 (66.5)					
	**Use of internet**												<.001	
		Yes	4376 (93.3)			2632 (97.9)			713 (91.1)					
		No	392 (6.7)			73 (2.1)			80 (8.9)					
**Medical history and disease condition**														
	**Ever had cancer**												<.001	
		Yes	1070 (15.3)			500 (12.7)			208 (20.8)					
		No	3538 (84.7)			2098 (87.3)			559 (79.2)					
	**General health status**												.02	
		Fair or poor	713 (14.3)			307 (10.8)			141 (16.3)					
		Good	1702 (36.6)			960 (37.1)			295 (35.3)					
		Excellent and very good	2256 (49.1)			1378 (52.2)			340 (48.4)					
	**Family history of cancer**												.53	
		Yes	3648 (83.2)			2093 (82.7)			593 (84.4)					
		No	647 (16.8)			366 (17.3)			105 (15.6)					
	**Depression or anxiety**												.40	
		No	3337 (29.3)			1833 (69.2)			587 (72.1)					
		Yes	1322 (70.7)			809 (30.8)			186 (27.9)					
	**Diabetes**												.049	
		Yes	908 (16.1)			458 (13.8)			160 (18.3)					
		No	3744 (83.9)			2181 (86.2)			612 (81.7)					
	**Hypertension**												.004	
		Yes	2034 (35.1)			1047 (32.4)			375 (41.2)					
		No	2621 (64.9)			1594 (67.6)			399 (58.8)					
	**Heart condition**												.77	
		Yes	460 (7.6)			220 (6.8)			73 (6.4)					
		No	4198 (92.4)			2422 (93.2)			701 (93.6)					
	**Chronic lung disease**												.53	
		Yes	694 (13.3)			394 (13.7)			115 (12.5)					
		No	3961 (86.7)			2247 (86.3)			658 (87.5)					
	**Number of disease conditions**												.10	
		None	2031 (51.7)			1227 (53.8)			326 (48.5)					
		One	1499 (30.2)			866 (30.1)			236 (30.6)					
		Two or more	1096 (18.1)			534 (16.1)			208 (20.9)					
**Lifestyle and behaviors**														
	**BMI**												.85	
		Underweight	234 (4.8)			116 (4.5)			36 (3.8)					
		Normal weight	1450 (31.8)			827 (33.1)			252 (31.7)					
		Overweight	1549 (31.3)			869 (30.3)			249 (32.8)					
		Obese	1537 (32.1)			893 (32.1)			256 (31.7)					
	**Physical activity**												.002	
		<150 min/wk	2948 (61.4)			1566 (57.7)			526 (70.3)					
		≥150 min/wk	1822 (38.6)			1139 (42.3)			267 (29.7)					

^a^Unweighted number of participants.

^b^Weighted percentages.

^c^Not applicable.

From the bivariate analysis, age, level of education, household income, use of the internet, history of cancer, reported health status, physical activity, hypertension, and diabetes status showed a significant association with the use of health and wellness apps (*P*<.05). More than two-thirds (85.1%) of participants within the age group 18-64 years used health and wellness apps compared to (14.9%) of participants within the age group 65+ years. A significant proportion of college graduates (n=1666, 48.2%) and individuals with household incomes of US $50,000 or more (n=1853, 77.2%) were found to use health and wellness apps. Among participants who use health and wellness apps, 87.3% (n=2098) had no history of cancer, 97.9% (2632) were internet users, 52.2% (n=1378) reported their general health status as excellent or very good, and 67.6% (n=1594) were hypertensive.

From the multivariable results in Table 2 and the final model (model 3), factors associated with the use of health and wellness apps included age, household income, use of the internet, higher educational level, and physical activity. We observed that as participant’s age increases, the odds of using health and wellness apps decrease. Participants within the age group 18-34 years were 4 times more likely (adjusted odds ratio [aOR]=3.70, 95% CI 1.90-7.23) to use a health and wellness app compared to participants within the age group of 75+ years. Compared to participants with household income below US $50,000, participants in the higher income category ≥US $50,000 had 49% higher odds of using mobile digital health and wellness apps (aOR=1.49, 95% CI 1.02-2.14).

Furthermore, consistent internet use significantly influenced the use of health and wellness apps across all 3 models. Internet users had 28% higher odds (aOR=3.28, 95% CI 1.70-6.33) of using mobile digital health and wellness apps compared to noninternet users. As the level of education increases, the odds of using health and wellness apps also increase. The participants with college graduate degrees and higher had 1.87 times the odds (95% CI 1.30-2.67) of using health or wellness apps compared to individuals with a high school diploma or less. Physical activity was the only lifestyle and behavioral factor found to be associated with the use of health and wellness apps. Compared to participants who engaged in less than 150 minutes of physical activity per week, those who exercised 150 minutes or more per week had 1.94 times higher adjusted odds (95% CI 1.40-2.70) of using health and wellness apps (Table 2).

**Table 2:** Bivariable and multivariable logistic regression analyses of the association between participant characteristics and the use of wellness and health apps^a^.

Characteristics			Used health and wellness app						
			Model 0^b^		Model 1^c^		Model 2^d^		Model 3^e^
			Crude OR^f^ (95% CI)		Adjusted OR (aOR; 95% CI)		Adjusted OR (aOR; 95% CI)		Adjusted OR (aOR; 95% CI)
**Age (years)**									
	18-34	*4.43 (2.75-7.14)*		*3.48 (1.97-6.16)*		*3.68 (1.9-7.1)*		*3.7 (1.9-7.23)*	
	35-49	*2.77 (1.72-4.46)*		*2.27 (1.31-3.94)*		*2.4 (1.29-4.45)*		*2.26 (1.22-4.2)*	
	50-64	*1.86 (1.21-2.87)*		1.49 (0.91-2.44)		1.68 (0.97-2.91)		1.63 (0.95-2.8)	
	65-75	1.21 (0.79-1.86)		1.12 (0.65-1.92)		1.18 (0.66-2.13)		1.07 (0.61-1.89)	
	75+	1		1		1		1	
**Gender at birth**									
	Female	1		1		1		1	
	Male	0.85 (0.66-1.08)		0.82 (0.63-1.07)		0.81 (0.62-1.07)		0.78 (0.6-1.03)	
**Insurance**									
	No	1		1		1		1	
	Yes	1.06 (0.64-1.74)		1.34 (0.72-2.49)		1.24 (0.62-2.47)		1.19 (0.58-2.46)	
**Education**									
	High school or less	1		1		1		1	
	Some college	1.34 (0.91-1.99)		1.2 (0.78-1.84)		1.23 (0.79-1.9)		1.19 (0.76-1.85)	
	College graduates and more	*2.26 (1.66-3.06)*		*1.84 (1.28-2.64)*		*1.95 (1.35-2.82)*		*1.87 (1.3-2.67)*	
**Household income (US $)**									
	< $50,000	1		1		1		1	
	≥$50,000	*1.71 (1.23-2.38)*		*1.47 (1.04-2.09)*		*1.49 (1.02-2.16)*		*1.49 (1.02-2.14)*	
**Use of internet**									
	No	1		1		1		1	
	Yes	*4.53 (2.63-7.79)*		*3.73 (1.97-7.08)*		*3.48 (1.75-6.93)*		*3.28 (1.70-6.33)*	
**Race or ethnicity**									
	Hispanic	1.16 (0.81-1.68)		1.32 (0.86-2.02)		1.41 (0.88-2.24)		1.4 (0.86-2.25)	
	Non-Hispanic Asian or non-Hispanic other	1.43 (0.9-2.27)		1.16 (0.73-1.86)		1.16 (0.7-1.93)		1.13 (0.67-1.89)	
	Non-Hispanic Black	1.05 (0.66-1.65)		1.3 (0.78-2.15)		1.2 (0.69-2.07)		1.18 (0.68-2.03)	
	Non-Hispanic White	1		1		1		1	
**General health status**									
	Fair or poor	1		N/A^h^		1		1	
	Good	*1.59 (1.1-2.30)*		N/A		1.28 (0.82-1.99)		1.3 (0.82-2.05)	
	Excellent and very good	*1.63 (1.15-2.32)*		N/A		1.23 (0.76-2)		1.2 (0.72-2.02)	
**Ever had cancer**									
	No	1		N/A		1		1	
	Yes	*0.55 (0.41-0.75)*		N/A		0.81 (0.57-1.15)		0.87 (0.62-1.23)	
**Family history of cancer**									
	No	1		N/A		1		1	
	Yes	0.88 (0.6-1.3)		N/A		0.9 (0.58-1.39)		0.86 (0.57-1.32)	
**Depression or anxiety**									
	No	1		N/A		1		1	
	Yes	1.15 (0.83-1.59)		N/A		0.92 (0.61-1.39)		0.93 (0.62-1.4)	
**Diabetes**									
	No	1		N/A		1		1	
	Yes	0.71 (0.51-1.01)		N/A		0.91 (0.4-2.12)		0.87 (0.37-2.07)	
**Hypertension**									
	No	1		N/A		1		1	
	Yes	*0.68 (0.53-0.89)*		N/A		0.77 (0.36-1.66)		0.8 (0.38-1.69)	
**Heart condition**									
	No	1		N/A		1		1	
	Yes	1.07 (0.69-1.67)		N/A		1.67 (0.94-2.95)		1.78 (1-3.17)	
**Chronic lung disease**									
	No	*1*		N/A		1		1	
	Yes	1.12 (0.79-1.57)		N/A		1.08 (0.55-2.11)		1.11 (0.57-2.17)	
**Number of disease conditions**									
	None	1		N/A		1		1	
	One	0.89 (0.65-1.22)		N/A		1.55 (0.74-3.26)		1.49 (0.71-3.13)	
	Two or more	*0.69 (0.52-0.94)*		N/A		1.46 (0.39-5.54)		1.38 (0.37-5.1)	
**BMI**									
	Underweight	1		N/A		N/A		1	
	Normal weight	0.89 (0.51-1.56)		N/A		N/A		0.65 (0.21-1.99)	
	Overweight	0.79 (0.44-1.43)		N/A		N/A		0.69 (0.21-2.22)	
	Obese	0.87 (0.47-1.61)		N/A		N/A		0.9 (0.27-3.05)	
**Physical activity**									
	<150 min/wk	1		N/A		N/A		1	
	≥150 min/wk	*1.73 (1.29-2.33)*		N/A		N/A		*1.94 (1.4-2.7)*	

^a^Italicized values are significant at *P*<.05.

^b^Model 0: univariate analysis.

^c^Model 1: sociodemographic factors.

^d^Model 2: sociodemographic factors + medical history and disease condition.

^e^Model 3: sociodemographic factors + medical history and disease condition + lifestyle and behavioral factors.

^f^OR: odds ratio.

^g^N/A: not applicable.

## Discussion

### Principal Findings

This study aimed to investigate the use of digital health and wellness apps among seekers of cancer information and identify factors predicting their use. Our results indicate a high prevalence of app usage among individuals seeking cancer information (81%), highlighting the potential of public health interventions to promote cancer prevention measures (for example, screening) and treatment through these platforms. Age and education emerged as the most significant predictors, with younger and more educated individuals showing a greater inclination toward the usage of these apps.

This finding aligns with similar studies [23,31], including one carried out among the Dutch population, which showed that younger age groups and individuals with higher education are more likely to use mHealth apps [32]. The reason may be that older patients generally describe themselves as not highly skilled in the use of mobile phones and other mobile devices [33]. In addition, older adults may face barriers such as a lack of trust in the apps, concerns about data privacy, and fear of misdiagnosis [34]. Moreover, other factors, such as age-related reduction in health functions such as memory, vision, and touch sensitivity, may hinder the effective use of mobile health apps [35]. The age disparity in mobile app use implies that older adults may experience worsened health outcomes and limited access to cancer health information due to lower engagement with digital tools. To address this, mobile apps can be designed to be more user-friendly for older adults, and digital health education programs should be implemented to encourage their adoption. Consistent with the Pew survey, which found that women were more likely to use health apps, gender differences were also observed in this study, with females being more likely to use health and wellness apps [36].

In a previous study, higher incomes were correlated with digital technology ownership and usage [37]. Our study found a similar association in which participants with household incomes greater than or equal to US $50,000 were more likely to use digital and wellness apps than participants with less than US $50,000. This suggests that income may strongly influence the use of digital and wellness apps even when other factors are considered. The finding that seekers of cancer information who met weekly recommendations for physical activity were more likely to use mHealth apps compared to those who engaged in less physical activity is consistent with previous studies [23,38]. This implies using health apps may enhance the achievement of physical activity health goals which has the potential to prevent chronic diseases [39].

Engaging in health information-seeking behavior, such as downloading mHealth apps, is acknowledged as a crucial activity during the “preparation stage” that may lead to changes in health behavior [40]. Use of the internet was found to be consistently associated with the usage of health and wellness apps among seekers of cancer information in our study. A similar study found that most internet users indicated a greater likelihood of using at least one eHealth tool to address a health issue over 12 months with a preference for YouTube videos, a peer-to-peer support website, or a smartphone app [41]. This implies that using the internet has the potential to empower individuals, enabling them to play a more active role in their health care and fostering changes in health information–seeking behavior by providing easy access to mHealth apps. Previous studies have shown that increased exposure to internet use and other web-based activities promotes the seeking of health information on the web [42]. The findings of our study confirm the impact of the internet on the use of health and wellness apps. Overall, our findings underscore the potential of mobile digital wellness and health apps in supporting cancer awareness and management.

### Clinical Practice Points and Implication

Technological interventions in cancer, specifically those using mobile digital wellness and health apps hold great promise to enhance patient outcomes and improve quality of life. Understanding factors that influence use is vital for creating impactful interventions. Apps should be developed to meet some specific health requirements such as phases of cancer treatment, as personalized intervention content is more likely to engage users and encourage behavioral changes, including social support features that allow users to communicate with caregivers, health care professionals, or individuals with similar health challenges will also go a long way toward heightening app usage and serve as a useful tool for interventions. Social support networks within the app can help users feel more connected and encouraged to share their experiences. Language and cultural differences should be considered to guarantee applicability and accessibility to a wide range of people. Enhanced engagement and effectiveness can also be achieved by providing content in multiple languages and taking cultural norms into account.

### Strengths

The major strength of our study was the comprehensive dataset we used. The samples used are fully representative of the noninstitutionalized US population, which enhances the generalizability of our results. Another strength of our study is its targeted population, specifically focusing on individuals who actively seek cancer information. This dataset was created by combining information from multiple survey cycles which boosted the sample size, thereby increasing the power and precision of our study. Additionally, the use of preexisting data minimized selection bias and allowed for proper comparisons with the existing literature. Finally, the consistency of our results with prior research further validates the reliability of our findings.

### Limitations

Despite the strengths of our study, it has limitations that warrant due consideration, the dataset used for our study was harmonized data from different HINTS cycles which were obtained from self-administered questionnaires. This method of data collection may introduce information bias, emphasizing the importance of more rigorous study designs in establishing causal relationships. Prospective studies and clinical trials are recommended to further explore the factors influencing digital health and wellness app usage among the general population and seekers of cancer information, and their impact on health outcomes among diverse populations. Additionally, our study did not take into account health literacy, provider recommendations, or health motivation. These factors should be considered in future research on the use of health and wellness apps. Another limitation of our study is the lack of data on the specific types of cancer information sought by participants. While this did not impact our goal of exploring factors influencing the use of mobile health and wellness apps among those seeking cancer information, there is a need for the HINT’s researchers to include a variable that focuses on the type of cancer information for further exploration by future studies.

### Conclusion

This study evaluated various factors that could impact the use of mobile health and wellness apps among cancer information seekers. Among the factors assessed; age, education, household income, use of the internet, history of cancer, and optimal health status were significantly linked to app usage. The results from this study suggest that individuals who have cancer could benefit remarkably from health apps, however, they were least likely to use the apps when compared to other factors. These apps may have the potential to address health care challenges, reduce disparities, and empower patients to manage their health more efficiently. Interventions can be tailored to enhance app use and improve health outcomes as we used retrospectively collected data in this cross-sectional study.

## Abbreviations

aOR: adjusted odds ratio
HINTS: Health Information National Trends Survey
mHealth: mobile health
STROBE: Strengthening the Reporting of Observational Studies in Epidemiology
WHO: World Health Organization

## Data Availability

The data access requests are publicly available on the Health Information National Trends Survey (HINTS) website. The code generated and used during this study is available upon request.
